# Initial symptoms and diagnostic delay in immune checkpoint inhibitor-related adrenal insufficiency: a systematic review and meta-ethnography of case reports

**DOI:** 10.3389/fendo.2025.1636452

**Published:** 2025-10-01

**Authors:** Ryuichi Ohta, Yoshinori Ryu, Kaoru Tanaka, Chiaki Sano, Hidetoshi Hayashi

**Affiliations:** ^1^ Department of Community Care, Unnan City Hospital, Unnan, Shimane, Japan; ^2^ Department of Medical Oncology, Kindai University Faculty of Medicine, Osakasayama, Osaka, Japan; ^3^ Community Medicine Management, Shimane University Faculty of Medicine, Izumo, Shimane, Japan

**Keywords:** immune checkpoint inhibitor, adrenal insufficiency, immune-related adverse event, diagnostic delay, fatigue, hypophysitis, case report, systematic review

## Abstract

**Background:**

Immune checkpoint inhibitors (ICIs) can cause adrenal insufficiency (AI) as an uncommon but potentially life-threatening immune-related adverse event (irAE). Early symptoms are often vague and may overlap with cancer-related fatigue or treatment side effects, contributing to diagnostic delays and under recognition.

**Methods:**

We conducted a systematic review and meta-ethnographic synthesis of 64 published case reports (2015–2024) describing ICI-associated AI. Data on demographics, tumor type, ICI regimen, presenting symptoms, diagnostic process, hormonal and imaging findings, treatment, and outcomes were extracted. Narrative data were synthesized to explore contextual barriers to timely diagnosis. Quality assessment was performed using a modified CARE checklist.

**Results:**

Fatigue (67.2%), nausea (28.1%), appetite loss (25.0%), and hypotension (21.9%) were the most frequently reported symptoms. Notably, most patients (73.7%) presented with three or more concurrent symptoms, suggesting a pattern of symptom clustering that warrants clinical attention. Despite this, only 40.6% underwent dynamic endocrine testing, and pituitary imaging was frequently normal. Meta-ethnographic analysis revealed five recurrent themes underlying diagnostic delay: nonspecific symptomatology, low clinical suspicion, delayed hormonal testing, misleading imaging, and fragmented specialty care. Hospitalization occurred in 60.9% of cases, and glucocorticoid therapy was initiated in all. Recovery of adrenal function was rare (9.4%).

**Conclusion:**

ICI-related adrenal insufficiency often presents with multiple nonspecific symptoms that are easily misattributed, delaying diagnosis. Clinicians should maintain a high index of suspicion, particularly when multiple symptoms co-occur in ICI-treated patients, and initiate prompt endocrine evaluation. Standardized diagnostic pathways and interdisciplinary communication are essential to improve recognition and outcomes of this serious irAE.

**Systematic review registration:**

https://www.crd.york.ac.uk/PROSPERO/view/, identifier CRD420251035850.

## 1 Introduction

Immune checkpoint inhibitors (ICIs) have revolutionized cancer immunotherapy and significantly improved survival outcomes in patients with various advanced solid tumors, including malignant melanoma and non-small cell lung cancer (1). However, the immune activation associated with ICIs can also lead to immune-related adverse events (irAEs), including endocrine toxicities such as thyroid dysfunction, hypophysitis, diabetes mellitus, and adrenal insufficiency (AI) (2, 3).

ICI-related AI includes two distinct entities. Central AI results from immune-related hypophysitis or isolated ACTH deficiency, typically without involvement of other anterior pituitary hormones. In contrast, primary AI arises from autoimmune adrenalitis, resulting in both glucocorticoid and mineralocorticoid deficiency, accompanied by markedly elevated ACTH and renin levels (4).

The incidence of central AI due to hypophysitis is relatively low with PD-1/PD-L1 inhibitors (0.4–1.0%) but increases significantly with CTLA-4 blockade (e.g., ipilimumab), such as ipilimumab (5–17%), making it the predominant endocrine toxicity of CTLA-4 blockade (5–7). Although large cohorts of hypophysitis have clarified the overall incidence and hormonal profiles, individual case reports remain valuable for characterizing delayed-onset AI, rare primary adrenalitis, and atypical presentations that are not captured in aggregated data (8, 9).

The initial symptoms of AI are typically nonspecific and may include fatigue, nausea, anorexia, weight loss, dizziness, hypotension, and altered consciousness (6). These symptoms can be easily misattributed to underlying cancer progression or the adverse effects of chemotherapy (7). Moreover, diagnostic evaluation, such as cortisol and ACTH testing or dynamic stimulation tests, is not always promptly performed in clinical practice, resulting in diagnostic delays that may increase morbidity and mortality (7).

Although several hypophysitis cohorts have described incidence, hormone profiles, and imaging findings, few studies have systematically analyzed the early clinical features of ICI-related AI or examined diagnostic delays. Furthermore, no previous review has comprehensively explored the patient-, clinician-, and system-level barriers to timely diagnosis. Understanding these factors is crucial for enhancing patient safety and facilitating early intervention in cancer immunotherapy.

## 2 Methods

### 2.1 Study design and registration

his systematic review and meta-ethnography were conducted following the PRISMA 2020 (Preferred Reporting Items for Systematic Reviews and Meta-Analyses) guidelines (8). The protocol was prospectively registered with the International Prospective Register of Systematic Reviews (PROSPERO; registration number: CRD420251035850).

### 2.2 Data sources and search strategy

We systematically searched PubMed, Embase, and Web of Science databases for articles published between January 1, 2000, and April 30, 2025. The search strategy combined terms related to immune checkpoint inhibitors (e.g., “immune checkpoint inhibitor,” “nivolumab,” “pembrolizumab,” “atezolizumab,” “ipilimumab”) with terms related to adrenal insufficiency (e.g., “adrenal insufficiency,” “Addison disease,” “hypopituitarism,” “isolated ACTH deficiency”) and study type (e.g., “case report,” “case series”). The full search strategy is available in the **Supplementary Material**. We included only English-language articles that involved human subjects and provided sufficient clinical detail. Additional studies were identified through manual reference list screening and citation tracking (“snowballing”).

### 2.3 Eligibility criteria

#### 2.3.1 Inclusion criteria

Studies were considered eligible for inclusion if they met all of the following criteria:

1. Study type: Published case reports or case series providing individual-level clinical data.
2. Population: Adult patients (aged ≥18 years) with a confirmed diagnosis of any malignancy.
3. Intervention: Treatment with at least one immune checkpoint inhibitor (ICI), including PD-1/PD-L1 inhibitors, or CTLA-4 blockade (e.g., nivolumab, pembrolizumab, atezolizumab, ipilimumab).
4. Attribution of adrenal insufficiency (AI): Explicit clinical attribution of adrenal insufficiency to ICI therapy, based on temporal association, exclusion of alternative causes, and/or immunologic plausibility.
5. Clinical detail: Sufficient documentation of at least one of the following domains:
  Initial presenting symptoms (e.g., fatigue, hypotension, anorexia)
  Endocrine laboratory findings (e.g., serum cortisol, ACTH levels)
  Imaging studies relevant to the hypothalamic-pituitary-adrenal axis (e.g., pituitary MRI)
  Diagnostic timeline (i.e., time from symptom onset to diagnosis)

Only English- or Japanese-language articles published between January 2015 and December 2024 were considered for inclusion.

#### 2.3.2 Exclusion criteria

The following were excluded from the review:

1. Insufficient clinical detail: Reports that lacked the minimum required clinical information to establish a diagnosis or assess the diagnostic process (e.g., missing symptom descriptions or hormonal results).
2. Unclear attribution: Cases in which adrenal insufficiency could not be reasonably linked to ICI therapy, due to alternative etiologies (e.g., brain metastases, infection, long-term corticosteroid use), or absence of causal inference by the authors.
3. Non-original reports: Review articles, editorials, expert opinions, or narrative overviews without primary patient-level data.
4. Abstract-only reports: Conference abstracts, posters, or oral presentations without accompanying full-text clinical detail.
5. Non-human studies: Experimental or preclinical studies involving animal models.
6. Duplications: Studies reporting overlapping cases without additional clinical detail or clarification.

### 2.4 Study selection

All references identified through the database search were imported into *Covidence* (Veritas Health Innovation, Melbourne, Australia), where duplicate records were automatically detected and removed. Two independent reviewers (R.O. and Y.R.) conducted the title and abstract screening in parallel. Articles deemed potentially eligible by either reviewer were retrieved for full-text evaluation.

The full-text review was also performed independently by the same two reviewers. Disagreements regarding study eligibility were resolved through discussion. A third reviewer (K.T.) was consulted to adjudicate if consensus could not be reached.

The study selection process was conducted in accordance with PRISMA 2020 guidelines and is presented as a PRISMA flow diagram, illustrating the number of records identified, screened, excluded, and included, along with reasons for exclusion at the full-text stage.

### 2.5 Data extraction

Data extraction was performed independently by two reviewers using a pre-defined and standardized extraction form developed for this review. The following variables were extracted from each included study:

- Patient demographics: age, sex
- Oncologic context: cancer type and stage
- ICI-related information: specific immune checkpoint inhibitor(s) administered (e.g., PD-1/PD-L1inihibitors, CTLA-4 blockade), monotherapy vs. combination regimen
- Clinical presentation: initial symptoms of adrenal insufficiency
- Timeline: interval from symptom onset to diagnosis
- Diagnostic evaluation: results of hormonal testing (e.g., cortisol, ACTH), dynamic endocrine testing, pituitary imaging findings (MRI/CT)
- Indicators of diagnostic delay: explicit or implied delays, including misdiagnosis, deferred testing, or missed clinical suspicion
- Management and outcomes: type of glucocorticoid replacement, need for hospitalization, recovery of endocrine function, resumption of ICI therapy, and tumor response

All extracted data were compiled into a centralized database and reviewed for internal consistency. Any discrepancies between reviewers were resolved by consensus after discussion. If necessary, clarification was obtained through re-review of the original full text.

### 2.6 Data synthesis and analysis

#### 2.6.1 Quantitative analysis

Descriptive statistics were employed to summarize both categorical and continuous variables extracted from the included case reports. Categorical data (e.g., frequencies and proportions of initial presenting symptoms, ICI agents used, types of adrenal insufficiency) were reported as counts and percentages. Continuous data (e.g., time from symptom onset to diagnosis) were reported as medians with accompanying ranges.

Data management, cleaning, and visual representation (e.g., frequency tables, bar graphs) were performed using Microsoft Excel and Python (pandas and matplotlib libraries). All analyses were exploratory and intended to characterize trends within the available case literature.

#### 2.6.2 Qualitative analysis

To synthesize narrative data relating to diagnostic delays, a meta-ethnographic approach was employed following the framework proposed by Noblit and Hare (9). The analysis proceeded through seven iterative phases: (1) identifying the focus of synthesis, (2) selecting relevant studies, (3) reading and re-reading texts, (4) determining key themes, (5) translating concepts across studies, (6) synthesizing translations, and (7) expressing the synthesis.

Thematic coding was performed using NVivo (QSR International), allowing for inductive identification of recurrent constructs relating to misdiagnosis, clinician decision-making, and system-level obstacles. A line-of-argument synthesis was used to construct a conceptual model describing how multilevel factors—including cognitive bias, diagnostic uncertainty, fragmented care, and inconsistent testing—interact to contribute to the delayed diagnosis of ICI-related adrenal insufficiency (10).

### 2.7 Quality assessment

Given the nature of the included studies (primarily single case reports and small case series), formal risk of bias tools such as the Newcastle-Ottawa Scale or ROBINS-I were not applicable. Instead, we performed a descriptive appraisal of reporting quality based on a modified CARE (Case Report) checklist. Key domains evaluated included: clarity of clinical timeline, completeness of endocrine evaluation, consideration of alternative diagnoses, availability and interpretation of imaging studies, and documentation of outcomes or follow-up. Summary statistics of reporting quality are provided in the Results section and visualized in Figure X.

## 3 Results

### 3.1 Study selection

A comprehensive search was conducted to identify published reports of ICI-related AI. Following database screening and eligibility assessment, 64 individual cases were included in this systematic review.

**Figure 1:**
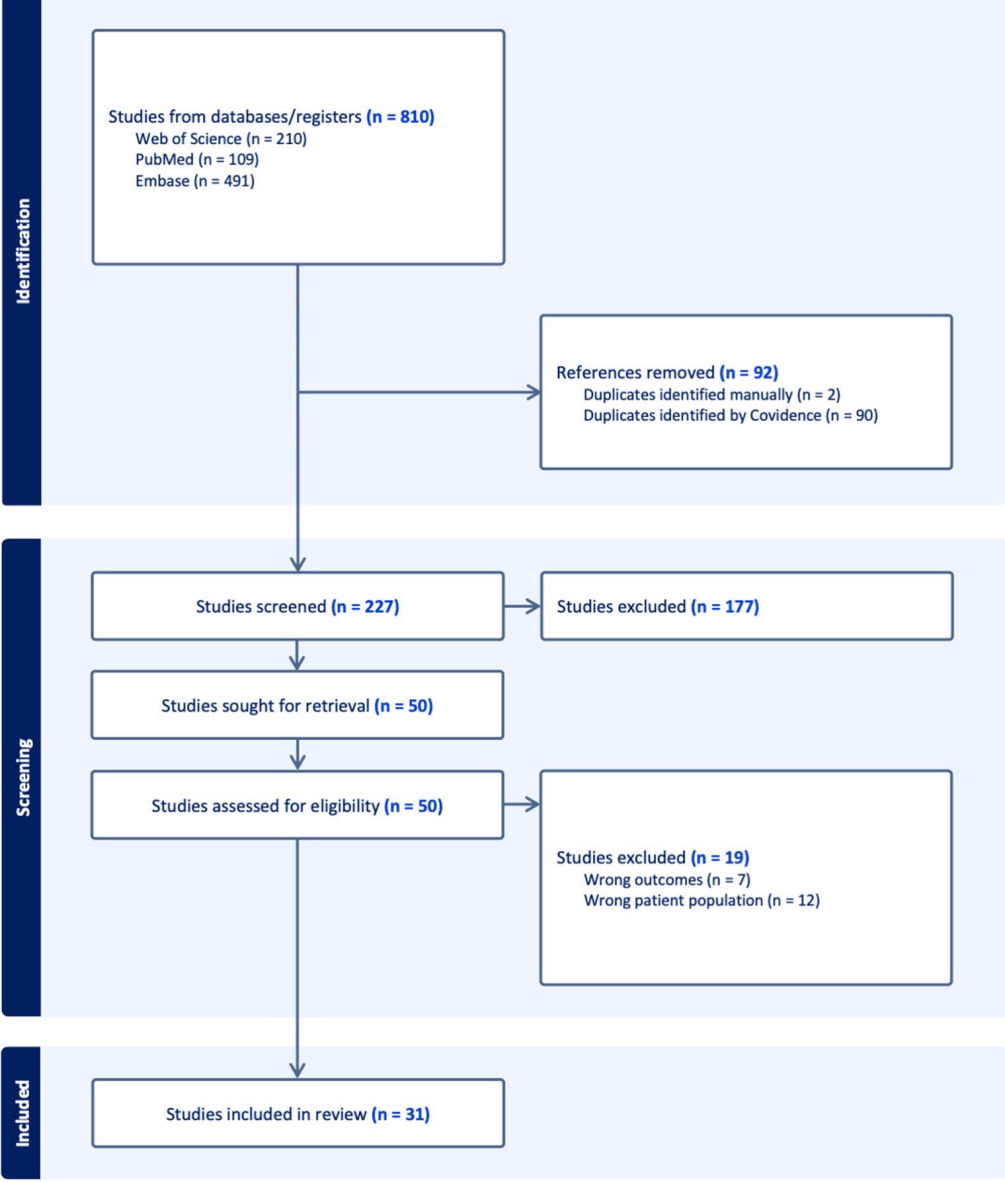
PRISMA (Preferred reporting items for systematic reviews and meta-analyses) 2020 flow diagram for new systematic reviews, which included searches of databases, registers and other sources.

The initial search yielded 810 studies. After removing duplicates and non-relevant titles and abstracts, 50 full-text articles were reviewed. 13 articles were excluded if they did not (1) describe adrenal insufficiency attributable to ICI therapy, (2) provide sufficient clinical or biochemical information to confirm the diagnosis, or (3) include individual case-level data. Thirty-seven studies comprising single case reports and series were included. All included cases were manually extracted and assessed for clinical features, diagnostic approach, imaging findings, treatment, and outcomes. A PRISMA flow diagram outlining the study selection process is presented in **Figure 1**.

### 3.2 Study cohort and case characteristics

A total of 64 cases of ICI-associated AI were identified from published case reports and series from 2015 to 2024. Most patients had advanced-stage solid tumors and received ICI therapy in the metastatic or unresectable setting (11–40) (**Supplementary Table S1**).

The most commonly reported primary malignancy was melanoma (n = 11, 17.2%). Other frequently observed cancers included non-small cell lung cancer (NSCLC) (n = 7, 10.9%), triple-negative breast cancer (n = 6, 9.4%), renal cell carcinoma (n = 4, 6.3%), hepatocellular carcinoma (n = 4, 6.3%), and ovarian cancer (n = 3, 4.7%). Less frequently reported tumor types included gastric cancer (n = 2), pancreatic cancer (n = 1), rectal cancer (n = 1), and periampullary carcinoma (n = 1), along with several cases from mixed or unspecified cohorts. ICIs were administered as monotherapy or in combination regimens across the cohort. Treatment indications reflected standard immunotherapy practices for advanced cancers during the respective study periods (**Table 1**).

**Table 1:** Summary of patient characteristics, treatment, adrenal insufficiency subtypes, and presenting symptoms in 64 published cases of ICI-associated AI.

Variable	n (%)
Total patients	64 (100)
Age, median (range)	64 (41–82)
Sex - Male	42 (65.6)
Sex - Female	22 (34.4)
Cancer type	
Melanoma	11 (17.2)
NSCLC	7 (10.9)
TNBC	6 (9.4)
Renal cell carcinoma	4 (6.3)
Hepatocellular carcinoma	4 (6.3)
Ovarian cancer	3 (4.7)
Others	29 (45.3)
ICI regimen	
PD-1/PD-L1 monotherapy	40 (62.5)
CTLA-4 blockade (ipilimumab)	8 (12.5)
Combination (PD-1 + CTLA-4)	16 (25.0)
Subtype of AI	
Central AI (hypophysitis)	59 (92.2)
Primary AI (autoimmune adrenalitis)	5 (7.8)
Onset timing	
During treatment	54 (84.4)
After discontinuation	10 (15.6)
Presenting symptoms	
Fatigue	43 (67.2)
Nausea	18 (28.1)
Appetite loss	16 (25.0)
Hypotension	14 (21.9)
Vomiting/diarrhea	10 (15.6)
Dizziness	9 (14.1)
Abdominal pain	6 (9.4)
Syncope	4 (6.3)
Number of symptoms	
Single symptom	6 (9.4)
Two symptoms	12 (18.8)
Three symptoms	26 (40.6)
Number of symptoms ≥ Four symptoms	20 (31.3)
ACTH recovery	6 (9.4)
ICI therapy resumed after AI	17 (26.6)

Age is presented as median (range). Cancer types include melanoma, non-small cell lung cancer (NSCLC), triple-negative breast cancer (TNBC), renal cell carcinoma (RCC), hepatocellular carcinoma (HCC), ovarian cancer, and other less common malignancies (e.g., gastric, pancreatic, rectal, periampullary carcinoma). ICI regimens include PD-1/PD-L1 inhibitors (nivolumab, pembrolizumab, atezolizumab), CTLA-4 blockade (ipilimumab), and combination therapy (e.g., nivolumab + ipilimumab). Central AI refers to secondary adrenal insufficiency due to immune-related hypophysitis or isolated ACTH deficiency (low/inappropriately normal ACTH with low cortisol).

Primary AI refers to autoimmune adrenalitis (elevated ACTH, low aldosterone, and high plasma renin activity).

Onset timing is categorized as during active ICI therapy or delayed onset after treatment discontinuation. Presenting symptoms are nonspecific clinical features at diagnosis; multiple symptoms were frequently clustered. Number of symptoms denotes the total symptom burden at presentation. ACTH recovery indicates documented partial or complete recovery of the hypothalamic–pituitary–adrenal axis after treatment. ICI therapy resumed after AI indicates that ICI treatment was safely restarted under endocrine supervision. Percentages (%) are calculated based on the total cohort (N = 64).

Importantly, the majority of cases (n = 59, 92.2%) represented central adrenal insufficiency (AI) secondary to immune-related hypophysitis or isolated ACTH deficiency, whereas only five cases (7.8%) fulfilled criteria for primary autoimmune adrenalitis with elevated ACTH, low aldosterone, and high plasma renin activity. This distinction highlights that central AI is the predominant phenotype in ICI-associated AI, particularly with CTLA-4 blockade, whereas primary adrenalitis remains rare.

### 3.3 Immune checkpoint inhibitors involved

Most cases involved PD-1 or PD-L1 inhibitors, either as monotherapy or in combination with CTLA-4 blockade. The most commonly implicated agents were nivolumab (23 cases), pembrolizumab (18 cases), ipilimumab (15 cases), and atezolizumab (10 cases). Less frequently reported agents included durvalumab (3 cases), tislelizumab (2 cases), camrelizumab (1 case), and avelumab (1 case) (**Table 1**). Combination regimens accounted for a notable proportion of cases. The most frequent combinations were ipilimumab plus nivolumab (7 cases) and atezolizumab plus bevacizumab (5 cases). Other complex regimens, including triplet combinations, were reported in isolated cases (**Table 1**).

### 3.4 Onset and clinical presentation

AI typically occurred between the second and twelfth ICI treatment cycle, with a median time to onset ranging from 4 to 6 months. Notably, 10 cases (15.6%) developed AI after discontinuation of ICI therapy, with onset ranging from 2 weeks to 8 months post-treatment, highlighting that delayed-onset AI can occur well beyond the active phase of immunotherapy.

The initial clinical presentation was often nonspecific. Fatigue was the most common symptom (n = 43, 67.2%), followed by nausea (18, 28.1%), appetite loss (16, 25.0%), hypotension (14, 21.9%), and anorexia (12, 18.8%). Vomiting and diarrhea were each reported in 10 cases (15.6%). Additional symptoms included dizziness (9), abdominal pain (6), and syncope (4). Rare but serious presentations included seizures (3) and altered mental status or disorientation (3), often occurring in the context of adrenal crisis (**Table 1**).

**Figure 2:**
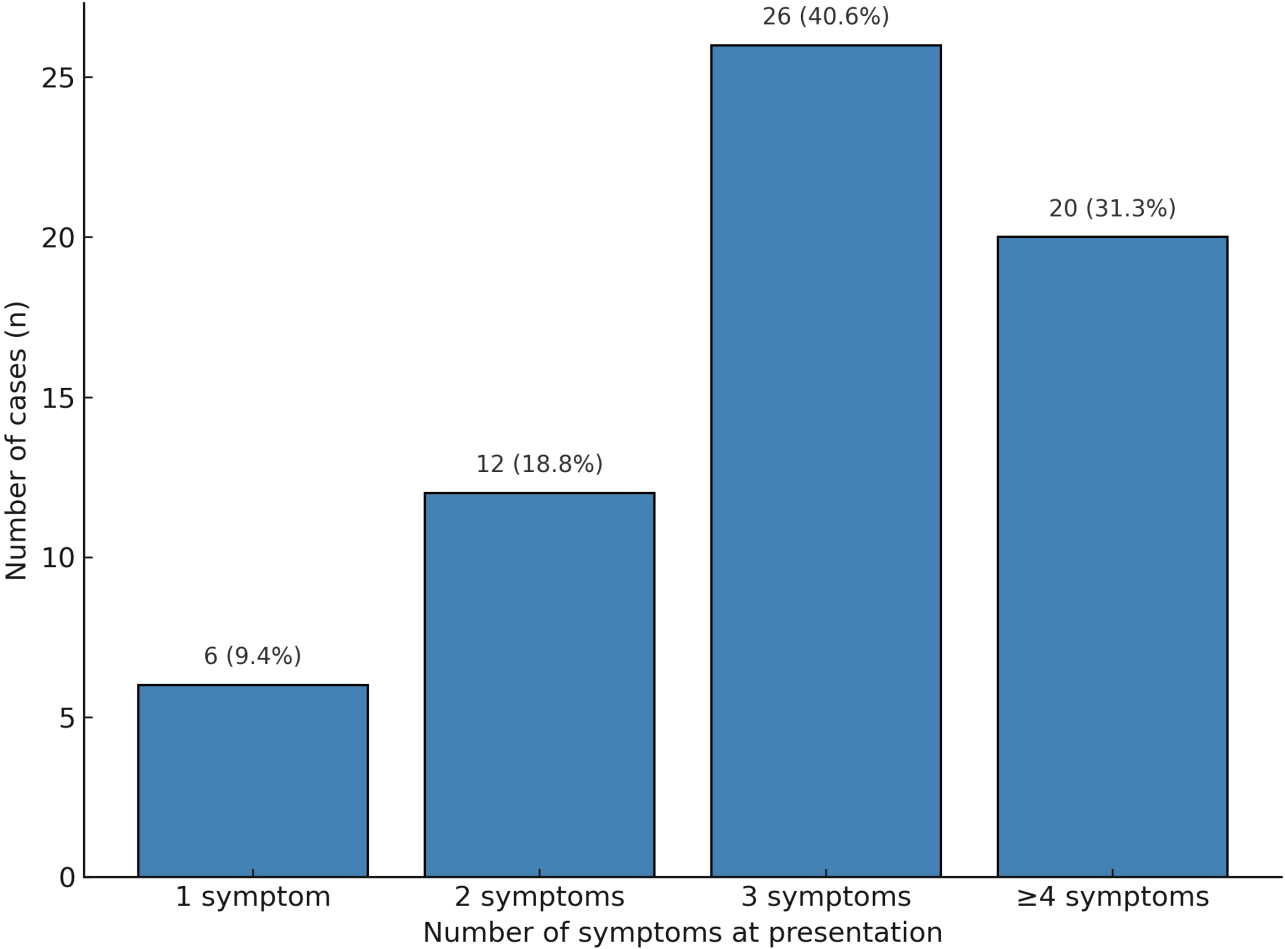
Distribution of the number of presenting symptoms in patients with ICI-associated adrenal insufficiency (n = 64).

When stratified by symptom burden, 6 patients (9.4%) presented with a single symptom, 12 patients (18.8%) had two symptoms, 26 patients (40.6%) had three symptoms, and 20 patients (31.3%) exhibited four or more concurrent symptoms. **Figure 2** was updated to clearly separate “three symptoms” and “≥ four symptoms” into distinct categories, rather than combining them. This demonstrates that most patients presented with clustered symptoms, which can still be misattributed to cancer-related fatigue or chemotherapy toxicity, contributing to diagnostic delay (**Table 1**).

### 3.5 Multiple clinical complaints

Most patients exhibited multiple clinical complaints when classified by the number of presenting symptoms. Specifically, 6 patients (9.4%) presented with a single symptom, 12 patients (18.8%) had two symptoms, 26 patients (40.6%) had three to four symptoms, and 20 patients (31.3%) exhibited five or more concurrent symptoms. These findings underscore the diagnostic complexity of ICI-associated AI, which frequently manifests with overlapping and nonspecific symptomatology (**Figure 2**).

### 3.6 Laboratory findings

Laboratory evaluation consistently supported the diagnosis of AI across the 64 cases included in this review. In nearly all reports, serum cortisol levels at presentation were significantly reduced, with 58 cases (90.6%) explicitly reporting values below 5 μg/dL—typically obtained in the early morning or during acute illness. These findings reflect the underlying disruption of the hypothalamic–pituitary–adrenal (HPA) axis.

Adrenocorticotropic hormone (ACTH) levels were available in 57 cases (89.1%). In the majority, ACTH levels were either suppressed or inappropriately normal despite low cortisol, a pattern characteristic of central AI, often resulting from immune-related hypophysitis. By contrast, five cases (7.8%) were consistent with primary AI, characterized by markedly elevated ACTH, low aldosterone, and high plasma renin activity, frequently accompanied by mucocutaneous hyperpigmentation.

Electrolyte abnormalities were frequently observed. Hyponatremia (serum sodium <130 mmol/L) was reported in 27 cases (42.2%), and eosinophilia was noted in 18 cases (28.1%), sometimes preceding clinical symptoms and serving as an early biochemical indicator of AI. Hypoglycemia was present in 12 cases (18.8%), most commonly in association with adrenal crisis or prolonged fasting.

Dynamic endocrine testing, such as corticotropin-releasing hormone (CRH) or ACTH stimulation testing, was inconsistently performed, reported in 26 cases (40.6%). These tests typically confirmed isolated ACTH deficiency. However, only a few cases included a comprehensive evaluation of additional anterior pituitary axes, highlighting that many reports lacked a full endocrine workup (**Table 2**).

**Table 2:** Key laboratory findings in reported cases of ICI-associated AI (n = 64).

Variable	n (%)
Morning cortisol < 5 μg/dL	58 (90.6)
ACTH level available	57 (89.1)
Suppressed/inappropriately normal ACTH (central AI)	52 (81.3)
Elevated ACTH (primary AI)	5 (7.8)
Hyponatremia (<130 mmol/L)	27 (42.2)
Eosinophilia (>500/μL)	18 (28.1)
Hypoglycemia (<70 mg/dL)	12 (18.8)
Dynamic endocrine testing performed	26 (40.6)
Confirmed isolated ACTH deficiency	24 (37.5)
Comprehensive anterior pituitary axis assessment	9 (14.1)
Pituitary imaging performed	62 (96.9)
Normal MRI/CT	48 (75.0)
Subtle pituitary changes (stalk thickening, partial empty sella, atrophy)	14 (21.9)
Adrenal imaging performed (suspected primary AI)	5 (7.8)

ACTH, adrenocorticotropic hormone; PAI, primary adrenal insufficiency; CRH, corticotropin-releasing hormone; µg/dL, micrograms per deciliter; mmol/L, millimoles per liter.

### 3.7 Diagnosis and radiographic findings

The diagnosis of ICI-associated AI was primarily established based on low morning serum cortisol concentrations, typically <4 μg/dL, combined with inappropriately low or normal adrenocorticotropic hormone (ACTH) levels. Among the 64 analyzed cases, dynamic endocrine testing was performed in 26 cases (40.6%), and isolated ACTH deficiency was confirmed in 25 (96.2%).

Pituitary imaging, typically via magnetic resonance imaging (MRI) or computed tomography (CT), was conducted in 62 patients (96.9%). Most scans (48 cases, 75.0%) revealed no abnormalities. However, mild structural changes were observed in 14 patients (21.9%), including stalk thickening, partial empty sella, or pituitary atrophy—findings suggestive of immune-related hypophysitis.

Notably, a routine CT scan cannot reliably exclude hypophysitis because of its limited sensitivity compared to a dedicated pituitary MRI; even an MRI can appear normal in early or isolated ACTH deficiency, leading to false reassurance.

Additionally, five cases (7.8%) met the primary AI criteria, evidenced by elevated ACTH levels, undetectable aldosterone, and high plasma renin activity, often accompanied by mucocutaneous hyperpigmentation (**Table 2**).

### 3.8 Treatment and outcomes

All patients received glucocorticoid replacement therapy. In most cases, intravenous hydrocortisone was initiated during the acute phase, followed by oral maintenance therapy (typically 10–20 mg/day). Hospitalization was required in 39 cases (60.9%), primarily due to adrenal crisis or hemodynamic instability.

However, contrary to earlier assumptions, most patients were not in adrenal crisis at the time of diagnosis and could be managed with oral hydrocortisone alone. Intravenous stress-dose hydrocortisone (e.g., 100 mg bolus followed by 50 mg every 6 hours) was reserved for patients with hypotension or altered consciousness. This aligns with SITC and NCCN guidelines, which state that stable adrenal insufficiency is not a contraindication to resuming ICI therapy.

Recovery of ACTH function was documented in 6 patients (9.4%), predominantly among those with primary adrenal involvement or transient central suppression.

These reversible cases suggest that in rare instances, hypophysitis-induced pituitary inflammation may be transient rather than destructive. More detailed reporting of these recovery cases is needed to distinguish factors predicting reversibility.

ICI therapy was resumed in 17 cases (26.6%), often under continued glucocorticoid replacement. Among the patients with available outcome data, 20 (31.3%) achieved objective tumor responses—complete or partial—suggesting that ICI therapy could be safely continued in selected cases following endocrine management (**Table 3**).

**Table 3:** Treatment approaches and clinical outcomes in patients with ICI-associated adrenal insufficiency (n = 64).

Parameter	Cases (n)	Percentage (%)
Glucocorticoid replacement administered	64	100%
Hospitalization required	39	60.9%
ACTH function recovery observed	6	9.4%
ICI therapy resumed	17	26.6%
Objective tumor response reported (CR or PR)	20	31.3%

ACTH, adrenocorticotropic hormone; ICI, immune checkpoint inhibitor; CR, complete response; PR, partial response.

### 3.9 Post-discharge follow-up

Among the 64 cases, follow-up data were available in 33 reports (51.6%). Recovery of adrenal function was rare, with only six cases (9.4%) showing evidence of recovery of the ACTH axis. Most patients required long-term glucocorticoid replacement therapy. Tumor outcomes were variably reported: 20 cases (31.3%) achieved partial or complete response. Notably, ICI therapy was resumed in 17 patients (26.6%), typically under endocrinologist supervision, with no reported cases of AI recurrence. However, long-term follow-up beyond six months was reported in only a minority of cases, limiting conclusions on chronic endocrine sequelae.

### 3.10 Meta-ethnography of diagnostic delay in ICI-associated adrenal insufficiency

#### 3.10.1 Getting started

This meta-ethnographic synthesis explored the mechanisms underlying diagnostic delay in patients who developed AI as an irAE following treatment with immune checkpoint inhibitors (ICIs). Drawing upon 64 published case reports and case series, the synthesis aimed to identify recurring patterns, explanatory constructs, and interpretive themes that may explain why delays in diagnosis occurred and how they could be mitigated in clinical practice.

#### 3.10.2 Determining relevance

All included reports were published between 2015 and 2024 and contained sufficient clinical detail to allow evaluation of the timeline from symptom onset to diagnostic confirmation. Each report provided some combination of initial symptoms, hormone testing results, imaging findings, timing of diagnosis, and therapeutic responses. These elements enabled the identification of implicit and explicit indicators of diagnostic delay.

#### 3.10.3 Reading the studies

The analysis involved close reading of each case to extract descriptions, clinical reasoning, and patterns indicative of diagnostic latency.

Recurrent observations included:

- Misattribution of early symptoms (e.g., fatigue, anorexia) to cancer progression or side effects of chemotherapy.
- Nonspecific and subtle clinical presentation, lacking hallmark endocrine features.
- Delays in ordering essential endocrine evaluations, such as serum cortisol and ACTH.
- Normal or unremarkable pituitary imaging, potentially leading to false reassurance.
- Fragmented care involves multiple departments without cohesive diagnostic coordination.

These recurring motifs provided the foundation for constructing second-order concepts and interpretive themes.

#### 3.10.4 Determining how the studies are related

Through constant comparison and coding, five dominant themes were identified as shared interpretive structures across the reports. These themes represented convergent clinical, cognitive, and system-level barriers contributing to diagnostic delay.

#### 3.10.5 Translation of studies into one another

Meta-ethnographic synthesis of 64 cases identified five recurring themes contributing to diagnostic delay in ICI-associated adrenal insufficiency. First, nonspecific symptoms—such as fatigue, nausea, and anorexia—were frequently misattributed to cancer progression or chemotherapy, as noted by Escaño et al. (28). Second, low clinical suspicion for endocrine irAEs, especially in monotherapy settings, led to delays, with diagnoses often made only after specialist referral (14). Third, delayed or omitted hormonal testing further impeded timely recognition (24). Fourth, normal pituitary MRI findings offered false reassurance, despite biochemical evidence of AI (30). Finally, fragmented care across emergency, oncology, and endocrinology services led to poor information continuity and overlooked treatment histories (11). These findings underscore the need for integrated care, early hormonal evaluation, and high suspicion when multiple nonspecific symptoms co-occur in ICI-treated patients.

#### 3.10.6 Synthesizing translations

The synthesis of translations revealed that diagnostic delay in ICI-induced AI is not merely the result of clinical ambiguity but stems from a confluence of cognitive, procedural, and systemic contributors. The non-specific nature of presenting symptoms, such as fatigue or nausea, is often misattributed to oncologic or chemotherapeutic causes. Moreover, the low index of suspicion for endocrine irAEs, particularly AI, appeared to reflect a broader gap in awareness among non-endocrine specialists.

Over-reliance on radiographic imaging, particularly MRI findings that were frequently normal or nonspecific in early hypophysitis, could provide false reassurance, leading clinicians to deprioritize endocrine testing. Furthermore, underutilizing hormonal assays such as morning cortisol, ACTH, and stimulation testing delayed definitive diagnosis. Fragmented care pathways, with poor communication among oncology, endocrinology, and emergency departments, further contributed to diagnostic inertia.

These findings supported a conceptual model in which diagnostic delay arises from the interaction of four domains: (1) patient-reported symptoms, (2) clinician interpretation, (3) diagnostic testing behavior, and (4) system-level care coordination. Interventions targeting all four levels—through clinician education, endocrine screening protocols, and integrated care pathways—may be essential to reduce diagnostic delays in future patients undergoing ICI therapy (**Table 4**).

**Table 4:** Themes with constructed insight, quote, and source.

Theme	Constructed insight	Quote	Source (author, year)
Nonspecific Symptomatology	Symptoms like fatigue, nausea, or anorexia were attributed to cancer progression or chemotherapy side effects in >50% of cases.	“Fatigue, nausea, generalized weakness, and hypotension … overlap significantly with other conditions, including the common side effects of cancer and its treatment, which resulted in an underestimation of our patient’s symptoms during prior admissions.”	Escaño et al., 2024 (28)
Lack of Awareness of irAE-Endocrinopathies	In many reports, clinicians did not initially suspect adrenal insufficiency as a potential immune-related event, particularly in mono-ICI cases.	The diagnosis was not considered until an endocrine consult was obtained, despite hypotension and hyponatremia.	Lin et al., 2019 (14)
Delayed Biochemical Evaluation	Delays in ordering ACTH and cortisol or misinterpreting borderline values contributed to diagnostic latency in over 30 cases.	Cortisol was not initially checked; instead, fluid resuscitation was prioritized.	Khatri et al., 2023 (24)
Unremarkable Imaging	Pituitary MRI was normal in 75.7% of cases, which may have falsely reassured clinicians against hypophysitis.	Although the MRI appeared normal, ACTH deficiency was confirmed by dynamic testing.	Kanie et al., 2018 (30)
Fragmented Care and Specialty Referral Gaps	Multiple cases involved transfer between oncology, endocrinology, and emergency departments, delaying definitive endocrine diagnosis.	“The observation unit team … discovered the patient was in active treatment with albumin-bound paclitaxel and atezolizumab, which was not noted in her electronic medical record.”	Yeung et al., 2024 (11)

### 3.11 Risk of bias within studies

Given that this systematic review was based exclusively on case reports and case series, the findings are inherently subject to a moderate to high risk of bias. Although such reports offer valuable insights into rare and heterogeneous presentations like ICI-associated AI, they often lack methodological rigor due to their descriptive and retrospective nature.

Several key domains of bias were observed across the 64 included cases:

Selection Bias: Many cases were published due to clinical severity or novelty—such as adrenal crisis, delayed onset, or atypical diagnostic features—raising concerns about publication bias. Asymptomatic or subclinical cases are likely underrepresented.

Diagnostic Heterogeneity: While all cases fulfilled biochemical criteria for AI, only 26 (40.6%) underwent dynamic endocrine testing (e.g., CRH or ACTH stimulation). Additionally, some reports’ pituitary imaging was incomplete or absent, complicating the distinction between central and primary AI.

Incomplete Reporting: Many reports lacked detailed clinical timelines, follow-up data, or rationale for excluding alternative diagnoses. ACTH and cortisol levels were often documented without corresponding assessment of aldosterone or renin, particularly in suspected cases of primary AI.

Lack of Standardized Outcome Assessment: Important clinical endpoints—such as hormonal recovery, resumption of ICI therapy, or tumor response—were variably reported. Only a minority of cases included follow-up beyond six months, limiting the ability to assess endocrine reversibility and long-term outcomes.

Quality Appraisal: Using a modified CARE (CAse REport) checklist, reporting quality varied considerably across studies:

Clinical timeline clearly documented: 60.9%
Comprehensive endocrine diagnostic panel: 45.3%
Differential diagnosis explicitly considered: 37.5%
Pituitary imaging findings described and interpreted: 70.3%
Longitudinal outcome or follow-up data included: 51.6%

**Figure 3:**
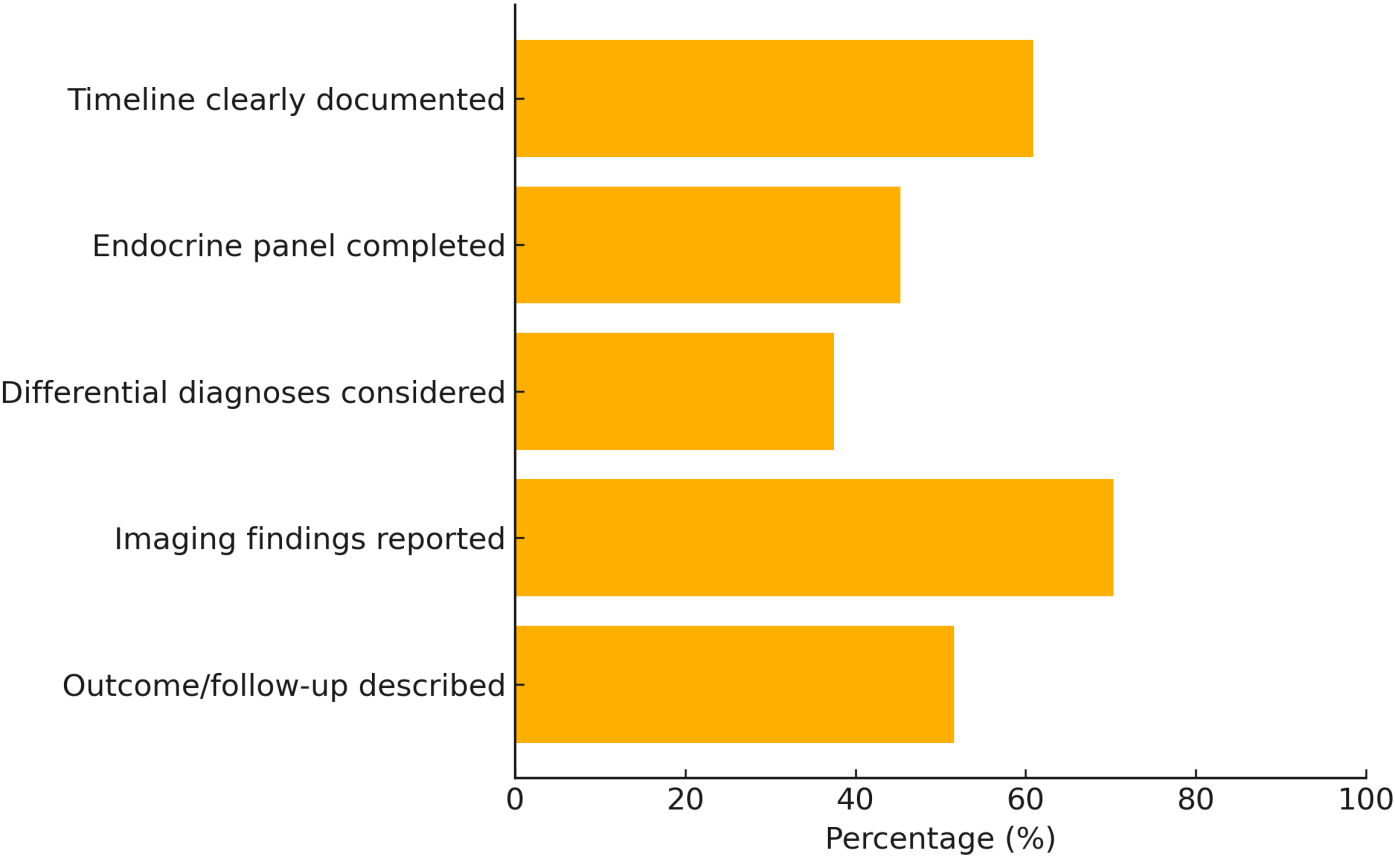
Quality of reporting among included case reports based on modified CARE checklist (n = 64).

In summary, while the reviewed reports contribute to our understanding of ICI-associated AI, substantial risk of bias remains due to case selection, diagnostic inconsistency, and incomplete reporting. These limitations highlight the urgent need for standardized reporting frameworks and prospective multicenter registries to enhance the validity and generalizability of future findings (**Figure 3**).

## 4 Discussion

### 4.1 Summary of main findings

This systematic review and meta-ethnographic synthesis analyzed 64 case reports and series of ICI-related AI published between 2015 and 2024. Our findings reveal that diagnostic delay is a common and multifactorial challenge in managing this rare but life-threatening irAE. Most cases presented with nonspecific symptoms such as fatigue, nausea, anorexia, and hypotension, which were frequently misattributed to cancer progression or treatment toxicity. Laboratory data consistently demonstrated low serum cortisol levels, with most cases reflecting central AI due to isolated ACTH deficiency. Despite the severity of symptoms in many cases, timely diagnosis was often hampered by clinical ambiguity, low clinician awareness of endocrine irAEs, and fragmented care pathways. Our meta-ethnography further synthesized these challenges into five major thematic domains, highlighting patient, clinician, diagnostic, and systemic contributors to diagnostic delay.

Importantly, this review clarifies that 92.2% of cases represented central AI secondary to hypophysitis, while only 7.8% fulfilled criteria for primary autoimmune adrenalitis. This supports prior observations that central AI is the predominant phenotype in ICI-associated AI, particularly with CTLA-4 blockade such as ipilimumab. Additionally, 15.6% of cases occurred after ICI discontinuation, indicating that delayed-onset AI can develop even beyond active treatment and necessitates long-term vigilance.

### 4.2 Comparison with previous literature

While the incidence of ICI-associated AI remains relatively low (0.4–1.0%), its clinical importance has been increasingly recognized in recent literature due to the potential for adrenal crisis and mortality if left untreated (4, 5).

However, this statement applies primarily to PD-1/PD-L1 monotherapy. With CTLA-4 blockade, particularly ipilimumab, hypophysitis is far more common, with reported incidence rates of 5–17% in melanoma cohorts (6–8). Our findings are consistent with these large hypophysitis cohorts, confirming that central AI with isolated ACTH deficiency is the most frequent endocrine phenotype, while primary adrenalitis remains rare.

Previous reviews and pharmacovigilance reports have primarily focused on incidence, hormone profiles, and endocrine outcomes (41–43), but few have addressed the diagnostic timeline or barriers in clinical recognition (44, 45). Our review builds upon this foundation by providing the most comprehensive synthesis to date of early presenting symptoms, delays in hormonal testing, and interdisciplinary gaps in care coordination.

Moreover, unlike prior hypophysitis cohorts that often lacked individual-level diagnostic timelines, our meta-ethnography highlights how symptom clustering, normal pituitary imaging, and fragmented specialty care create a “diagnostic inertia” that delays recognition of AI. This nuanced understanding complements existing cohort studies by explaining *why* diagnostic delays persist even when clinicians are aware of endocrine irAEs.

### 4.3 Clinical implications

Clinically, this review underscores the importance of maintaining a high index of suspicion for AI in ICI-treated patients who present with unexplained fatigue, hypotension, nausea, or mental status changes. As most cases involved central AI, often without pituitary imaging abnormalities, reliance on radiographic findings alone is insufficient for exclusion (46).

A normal CT scan should not be considered sufficient to rule out hypophysitis because of its poor sensitivity compared with dedicated pituitary MRI. Even MRI may appear normal in early or isolated ACTH deficiency, leading to false reassurance (47). Hence, timely hormonal testing (morning cortisol, ACTH, and dynamic testing) remains essential for early diagnosis.

Treatment typically consisted of glucocorticoid replacement, with intravenous hydrocortisone often initiated in the acute phase followed by oral maintenance therapy.

However, our findings indicate that most patients were not in adrenal crisis at the time of diagnosis and were successfully stabilized with oral hydrocortisone alone. Intravenous stress dosing was reserved for patients with hypotension or altered mental status. Moreover, stable AI is not a contraindication to continuing or resuming ICI therapy (48). In fact, 17 patients (26.6%) in our review safely resumed immunotherapy under endocrine supervision, which is consistent with SITC and NCCN guidelines.

### 4.4 Unique findings: recovery of ACTH axis

A notable finding in this review was that six patients (9.4%) showed recovery of ACTH function. This suggests that in rare cases, hypophysitis-induced pituitary inflammation may be transient and reversible rather than permanently destructive. This expands the known spectrum of immune-mediated hypophysitis and highlights the need for prospective studies to investigate predictors of reversibility, such as early diagnosis, prompt steroid replacement, and immune-modulatory factors.

### 4.5 Why case reports remain relevant

Although hypophysitis is no longer considered rare with CTLA-4 blockade, individual case reports remain valuable for several reasons. First, delayed-onset AI after ICI discontinuation is still under-recognized and often captured only in case-based literature. Second, rare primary adrenalitis cases with 21-hydroxylase antibodies provide mechanistic insight into adrenal autoimmunity. Third, unusual symptom clusters (e.g., seizures, abdominal pain) and reversible ACTH recovery cases expand the clinical phenotype beyond what large cohorts capture. Therefore, case reports continue to complement cohort studies by identifying novel or atypical presentations.

### 4.6 Limitations

This study also highlights limitations in current reporting practices. Many cases lacked full endocrine panels, dynamic testing, or clear diagnostic timelines, limiting comparability. Moreover, larger hypophysitis cohorts could not be included because they did not provide individual-level clinical detail required for meta-ethnographic synthesis. This introduces potential selection bias toward more severe or atypical cases. Nonetheless, our findings remain complementary to these cohorts by focusing on diagnostic delays and patient-level symptom trajectories. Future efforts should focus on standardized case reporting following CARE guidelines (48) and on establishing prospective endocrine registries to systematically capture onset timing, hormonal data, imaging findings, and outcomes.

### 4.7 Future directions

Moreover, our meta-ethnographic model offers a conceptual framework that can guide future research into system-based interventions, such as clinical decision support tools, early warning systems, and multi-disciplinary care algorithms to facilitate earlier recognition of ICI-related endocrinopathies. Additionally, incorporating routine endocrine screening (e.g., periodic morning cortisol) into ICI protocols, particularly for high-risk regimens like ipilimumab, may facilitate earlier detection of subclinical or asymptomatic AI. Furthermore, long-term post-ICI surveillance is needed to capture delayed-onset cases, which represented 15.6% in our cohort. Finally, prospective studies should investigate predictors of ACTH axis recovery to identify patients who may not require lifelong steroid replacement.

### 4.8 Conclusion

In summary, our findings integrate and extend existing literature by clarifying that central AI is the predominant form of ICI-related AI, highlighting diagnostic delays due to nonspecific symptoms and normal imaging, and showing that stabilized AI does not preclude ongoing immunotherapy. By complementing large hypophysitis cohorts with granular case-level data, this review identifies actionable diagnostic barriers and underscores the ongoing relevance of case reports for rare phenotypes, delayed-onset AI, and reversible pituitary dysfunction.

## Data Availability

The original contributions presented in the study are included in the article/**Supplementary Material**. Further inquiries can be directed to the corresponding author.
