# Supplementary material for: Initial symptoms and diagnostic delay in immune checkpoint inhibitor-related adrenal insufficiency: a systematic review and meta-ethnography of case reports
Source: Front Endocrinol (Lausanne). 2025 Oct 1;16:1636452. doi: 10.3389/fendo.2025.1636452 (PMC12520904; doi:10.3389/fendo.2025.1636452)
Supplement: Supplementary file 1 [file Table1.docx]

Supplementary Table 1

| Author/Year | Cancer Type | ICI Used | Onset Timing | Initial Symptoms | Diagnosis Method | Severity (CTCAE) | Treatment | Outcome | Notes |
| --- | --- | --- | --- | --- | --- | --- | --- | --- | --- |
| Yeung et al., 2024 [11] | Triple-negative breast cancer (TNBC) | Pembrolizumab | After 3 cycles; last dose 10 days prior | Fatigue, nausea, dizziness | Cortisol <0.87 μg/dL, ACTH <2 pg/mL, neutropenic fever | Not explicitly stated; treated as adrenal crisis | IV fluids, antibiotics, stress-dose hydrocortisone | Stabilized, discharged with hydrocortisone | Central adrenal insufficiency likely due to hypophysitis |
| Yeung et al., 2024 [11] | Oropharyngeal squamous cell carcinoma | Pembrolizumab (20 cycles) | Symptoms started 6 days before 1st ED visit; diagnosed on 2nd visit | Diarrhea, fatigue, arm pain | Cortisol 1.83μg /dL, ACTH <2 pg/mL, MRI: partially empty sella | Not stated | Hydrocortisone 50 mg IV q8h | Discharged with hydrocortisone and opioids | Central adrenal insufficiency, MRI showed partially empty sella |
| Yeung et al., 2024 [11] | PR+ breast cancer (HER2-) | Atezolizumab + nab-paclitaxel | 1 day after symptom onset, 3 days after COVID booster | Nausea, vomiting, weakness, hypoglycemia | Cortisol <0.87μg /dL, ACTH <2 pg/mL, CRP, ESR elevated | Not stated | Stress-dose hydrocortisone IV | Improved, discharged with hydrocortisone | Consistent with hypophysitis-induced adrenal insufficiency |
| Yeung et al., 2024 [11] | Triple-negative breast cancer (TNBC) | Pembrolizumab + chemotherapy | 4 days after last dose | Fatigue, nausea | Cortisol and ACTH undetectable | Not stated | Hydrocortisone 50 mg IV | Discharged with hydrocortisone | Planned for surgery, diagnosis made early due to pre-op workup |
| Yeung et al., 2024 [11] | Triple-negative breast cancer (TNBC) | Pembrolizumab + carboplatin + paclitaxel | 35 days after last pembrolizumab | Nausea, vomiting, fatigue, headache, gait instability | Cortisol <0.87μg /dL, ACTH 3 pg/mL, poor cosyntropin response | Not stated | Hydrocortisone replacement therapy | Improved, discharged in 2 days | Likely mixed central and primary adrenal insufficiency |
| Han et al., 2022 [12] | Non-small-cell lung carcinoma (NSCLC) | Camrelizumab | After 11 cycles (~22 weeks) | General malaise, appetite loss | Low cortisol, low ACTH, low TSH; MRI: partial empty sella | Not stated | Methylprednisolone +hydrocortisone maintenance | Improved, ICI discontinued | Central adrenal insufficiency, MRI showed partial empty sella |
| Han et al., 2022 [12] | Hepatocellular carcinoma (HCC) | Camrelizumab + Apatinib | After 10 cycles | Syncope, hypotension, fever | Very low cortisol and ACTH, low TSH; MRI normal | Not stated, adrenal crisis suggested | Methylprednisolone +prednisone taper | Recovered, ICI discontinued | ACTH and cortisol deficiency remained; TSH recovered |
| Han et al., 2022 [12] | Clear cell renal cell carcinoma (CCRCC) | Camrelizumab + Apatinib | After 8 cycles (~16 weeks) | Appetite loss, fatigue, diarrhea | Low cortisol, undetectable ACTH; MRI normal | Not stated | Methylprednisolone, hydrocortisone maintenance | Recovered | Secondary adrenal insufficiency, testosterone low |
| Cheng et al., 2024 [13] | Triple-negative breast cancer (TNBC) | Pembrolizumab | Approximately 4 months after initiation | Weakness, hypotension, tachycardia, tachypnea | Cortisol <0.5μg /dL, ACTH <1.5 pg/mL, poor response to cosyntropin test | Not formally graded; presented with recurrent hypotension | Hydrocortisone, fludrocortisone, midodrine, transitioned to hydrocortisone | Stable on physiologic dose glucocorticoid | Pituitary MRI not done due to breast tissue expander; secondary adrenal insufficiency |
| Lin et al., 2019 [14] | Lung adenocarcinoma | Nivolumab | 7th cycle / 14 weeks | Hypotension, nausea, vomiting | Low 8AM cortisol, low ACTH | Grade 3 | Prednisolone 15 mg/day | Stable disease | MRI normal |
| Lin et al., 2019 [14] | Urothelial carcinoma | Anti-CTLA-4 + Anti-PD-L1 | 4th cycle / 16 weeks | Lethargy | Low ACTH, low cortisol, primary hypothyroidism | Grade 3 | Prednisolone 5 mg/day + Levothyroxine 50 mcg/day | Expired | Confirmed with dynamic testing |
| Lin et al., 2019 [14] | Melanoma | Pembrolizumab | 8th cycle / 36 weeks | Headache, poor appetite, fatigue | Low ACTH, low cortisol, thyroid dysfunction | Grade 3 | Prednisolone 7.5 mg/day + Levothyroxine 50 mcg/day | Progressive disease | MRI not available |
| Lin et al., 2019 [14] | Lung adenocarcinoma | Anti-PD-1 based combination | 3rd cycle / 5 weeks | None reported | Low ACTH and cortisol, Graves' disease | Grade 1 | Prednisolone 5 mg/day | Expired | Discontinued steroids after 3 months |
| Lin et al., 2019 [14] | Periampullary adenocarcinoma | Pembrolizumab | 5th cycle / 30 weeks | Poor appetite | Low ACTH, low cortisol, low testosterone | Grade 2 | Cortisone 37.5 mg/day | Progressive disease | Topical testosterone initiated |
| Lin et al., 2019 [14] | Cervical squamous cell carcinoma | Anti-CTLA-4 + Anti-PD-1 →Anti-PD-1 alone | 13th cycle / 33 weeks | Fatigue, weight loss | Low ACTH, low cortisol, thyroid dysfunction | Grade 2 | Cortisone 37.5 mg/day + Levothyroxine 50 mcg/day | Stable disease | No pituitary MRI abnormality |
| Lin et al., 2019 [14] | Lung sarcomatoid carcinoma | Pembrolizumab | 5th cycle / 27 weeks | Poor appetite, weight loss | Low ACTH and cortisol | Grade 2 | Prednisolone 7.5 mg/day | Progressive disease | MRI: less brightness posterior lobe |
| Lupi et al., 2019 [15] | NSCLC | Atezolizumab (anti-PD-L1) | After 4th dose | Hyponatremia, hyperkalemia, DKA (T1DM) | Cortisol <0.4μg /dL, ACTH 4 ng/L, MRI normal, positive pituitary & adrenal Abs | Not reported; adrenal crisis suggested | Hydrocortisone, fludrocortisone, insulin; ICI discontinued | Tumor progression at 3 months | Autoimmune polyendocrine syndrome, positive 21-hydroxylase Ab |
| Lupi et al., 2019 [15] | NSCLC | Nivolumab (anti-PD-1) | After 6th cycle | Nausea, vomiting, fatigue, drowsiness | Cortisol <3μg /dL, ACTH <5 ng/L, MRI normal | Not reported | Hydrocortisone: ICI discontinued | Tumor progression at 3 months | Central adrenal insufficiency with hypogonadism |
| Lupi et al., 2019 [15] | Melanoma | Pembrolizumab (anti-PD-1) | After 9 cycles (2+7), ~6 months | Headache, muscle weakness | Cortisol 0.4μg /dL, ACTH <5 ng/L, MRI normal | Not reported | Hydrocortisone, continued pembrolizumab | No tumor progression at 6 months | Isolated ACTH deficiency |
| Lupi et al., 2019 [15] | Melanoma | Nivolumab or ipilimumab + nivolumab | 7 days after combination therapy started | Fever, profound asthenia | Cortisol 0.8μg /dL, ACTH 5 ng/L, MRI normal | Not reported | Hydrocortisone, continued insulin, L-thyroxine, and ICI | No tumor progression at 6 months | Multiple autoimmune irAEs, including T1DM and vitiligo |
| Percik et al., 2020 [16] | Melanoma | Pembrolizumab | 16 months after ICI initiation | Fatigue, anorexia, weight loss | Low cortisol + low/inappropriate ACTH, intact other pituitary axes | Not reported; some with hypotension or hyponatremia | Physiologic glucocorticoids (prednisone 5 mg/day) | Symptomatic improvement, continued ICI in all | Hypotension at diagnosis |
| Percik et al., 2020 [16] | Melanoma | Nivolumab + Ipilimumab | 3 months after ICI initiation | Fatigue, nausea/vomiting, diarrhea | Low cortisol + low/inappropriate ACTH, intact other pituitary axes | Not reported; some with hypotension or hyponatremia | Physiologic glucocorticoids (prednisone 5 mg/day) | Symptomatic improvement, continued ICI in all | Hyponatremia, eosinophilia |
| Percik et al., 2020 [16] | Ovary | Durvalumab + Olaparib | 7 months after ICI initiation | Fatigue, anorexia, nausea/vomiting, myalgia | Low cortisol + low/inappropriate ACTH, intact other pituitary axes | Not reported; some with hypotension or hyponatremia | Physiologic glucocorticoids (prednisone 5 mg/day) | Symptomatic improvement, continued ICI in all |  |
| Percik et al., 2020 [16] | Melanoma | Nivolumab + Ipilimumab | 9 months after ICI initiation | Fatigue, nausea/vomiting, myalgia | Low cortisol + low/inappropriate ACTH, intact other pituitary axes | Not reported; some with hypotension or hyponatremia | Physiologic glucocorticoids (prednisone 5 mg/day) | Symptomatic improvement, continued ICI in all | Eosinophilia; thyroiditis, arthritis |
| Percik et al., 2020 [16] | Melanoma | Pembrolizumab → Nivolumab → Nivolumab + Ipilimumab | 8 months after ICI initiation | Fatigue, anorexia, nausea/vomiting | Low cortisol + low/inappropriate ACTH, intact other pituitary axes | Not reported; some with hypotension or hyponatremia | Physiologic glucocorticoids (prednisone 5 mg/day) | Symptomatic improvement, continued ICI in all | Thyroiditis, nephritis, encephalitis |
| Percik et al., 2020 [16] | Breast | Pembrolizumab + Paclitaxel | 6 months after ICI initiation | Fatigue, diarrhea, myalgia | Low cortisol + low/inappropriate ACTH, intact other pituitary axes | Not reported; some with hypotension or hyponatremia | Physiologic glucocorticoids (prednisone 5 mg/day) | Symptomatic improvement, continued ICI in all | Pneumonitis |
| Percik et al., 2020 [16] | Renal | Nivolumab + Ipilimumab | 3 months after ICI initiation | Fatigue, weight loss, diarrhea | Low cortisol + low/inappropriate ACTH, intact other pituitary axes | Not reported; some with hypotension or hyponatremia | Physiologic glucocorticoids (prednisone 5 mg/day) | Symptomatic improvement, continued ICI in all | Hyponatremia, eosinophilia |
| Percik et al., 2020 [16] | Melanoma | Pembrolizumab → Ipilimumab + Nivolumab | 6 months after ICI initiation | Fatigue, anorexia | Low cortisol + low/inappropriate ACTH, intact other pituitary axes | Not reported; some with hypotension or hyponatremia | Physiologic glucocorticoids (prednisone 5 mg/day) | Symptomatic improvement, continued ICI in all | Hyponatremia |
| Percik et al., 2020 [16] | Gastric | Nivolumab + Ipilimumab | 3 months after ICI initiation | Fatigue, anorexia | Low cortisol + low/inappropriate ACTH, intact other pituitary axes | Not reported; some with hypotension or hyponatremia | Physiologic glucocorticoids (prednisone 5 mg/day) | Symptomatic improvement, continued ICI in all | Hyponatremia |
| Percik et al., 2020 [16] | Transitional Cell Carcinoma | Pembrolizumab + Gemcitabine + Cisplatin | 6 months after ICI initiation | Fatigue, anorexia, diarrhea, myalgia, depression | Low cortisol + low/inappropriate ACTH, intact other pituitary axes | Not reported; some with hypotension or hyponatremia | Physiologic glucocorticoids (prednisone 5 mg/day) | Symptomatic improvement, continued ICI in all | Hypotension, hyponatremia |
| Percik et al., 2020 [16] | Melanoma | Nivolumab + Ipilimumab → Pembrolizumab | 13 months after ICI initiation | Fatigue, anorexia, weight loss, nausea/vomiting, diarrhea | Low cortisol + low/inappropriate ACTH, intact other pituitary axes | Not reported; some with hypotension or hyponatremia | Physiologic glucocorticoids (prednisone 5 mg/day) | Symptomatic improvement, continued ICI in all | Hypotension, hyponatremia; thyroiditis |
| Percik et al., 2020 [16] | Melanoma | Ipilimumab | 4 months after ICI initiation | Fatigue, anorexia, nausea/vomiting, diarrhea | Low cortisol + low/inappropriate ACTH, intact other pituitary axes | Not reported; some with hypotension or hyponatremia | Physiologic glucocorticoids (prednisone 5 mg/day) | Symptomatic improvement, continued ICI in all | Colitis |
| Percik et al., 2020 [16] | Ovary | Pembrolizumab + anti-TIGIT | 4 months after ICI initiation | Fatigue, anorexia | Low cortisol + low/inappropriate ACTH, intact other pituitary axes | Not reported; some with hypotension or hyponatremia | Physiologic glucocorticoids (prednisone 5 mg/day) | Symptomatic improvement, continued ICI in all | Hyponatremia, eosinophilia |
| Percik et al., 2020 [16] | Melanoma | Nivolumab + Ipilimumab | 4 months after ICI initiation | Fatigue, anorexia, nausea/vomiting, diarrhea | Low cortisol + low/inappropriate ACTH, intact other pituitary axes | Not reported; some with hypotension or hyponatremia | Physiologic glucocorticoids (prednisone 5 mg/day) | Symptomatic improvement, continued ICI in all | Hyponatremia |
| Thapi et al., 2019 [17] | Clear cell renal cell carcinoma (CCRCC) | Nivolumab →Nivolumab + Ipilimumab | 3 weeks after starting combination therapy | Sudden severe fatigue, cold intolerance | Cortisol 2.0μg /dL (1 pm), ACTH 16 pg/mL, MRI normal | Not stated; initial high-dose steroids used | Prednisone 1 mg/kg → tapered to 5 mg → discontinued | Full recovery of HPA axis (cortisol 13.0 ﾎｼg/dL off steroids) | Extremely rare case of reversible secondary adrenal insufficiency from ICI |
| Chang et al., 2019 [18] | Non-small cell lung cancer (NSCLC) | Nivolumab | Approximately 1 year after initiation | Fatigue, weight loss, emesis, tachycardia, dry mucosa | Cortisol 5.6μg /dL (AM), ACTH 8 pg/mL, poor response to cosyntropin, MRI: partially empty sella | Not formally graded; consistent with central adrenal insufficiency | Hydrocortisone (20+10 mg/day) → prednisone taper to 15 mg/day | Clinically stabilized; follow-up endocrinology planned | Hypopituitarism (adrenal insufficiency and hypogonadism), partially empty sella consistent with late-stage hypophysitis |
| Takeno et al., 2019 [19] | Malignant melanoma | Nivolumab (2 mg/kg every 3 weeks) | 6 months after discontinuation of ICI (17 months after initiation) | General malaise, appetite loss, hyponatremia, hypoglycemia | ACTH 9.6 pg/mL, undetectable cortisol, no response to CRH, MRI/CT normal | Not stated; severe hyponatremia/hypoglycemia | Hydrocortisone replacement | Symptoms and labs improved | Delayed-onset isolated ACTH deficiency; highlights need for monitoring after ICI discontinuation |
| Otsubo et al., 2017 [20] | Lung adenocarcinoma (NSCLC) | Nivolumab (2 doses) | 4 months after discontinuation | Fatigue, appetite loss, eosinophilia (23.8%) | Cortisol 0.5 mg/dL, ACTH 2.4 pg/mL, MRI normal | Not formally stated; consistent with central AI | IV methylprednisolone pulse → oral hydrocortisone 15 mg/day | Symptoms and eosinophilia improved | No pituitary enlargement; adrenal insufficiency occurred post-discontinuation |
| Otsubo et al., 2017 [20] | Lung adenocarcinoma (NSCLC) | Nivolumab (11 cycles) | 6 months after discontinuation | Fatigue, diarrhea, eosinophilia (38.6%) | Cortisol 1.0 mg/dL, ACTH <1.0 pg/mL, MRI normal | Not stated; secondary AI with severe eosinophilia | IV hydrocortisone → oral hydrocortisone (tapered from 40 mg/day) | Clinical improvement and eosinophil normalization | Emphasized need for monitoring even post-ICI withdrawal |
| Hinata et al., 2021 [21] | Urothelial carcinoma with ileal conduit | Pembrolizumab | 4.5 months after initiation | Anorexia, general weakness, muscle pain, hyperkalemia, metabolic acidosis | Cortisol 1.4μg /dL, ACTH 16.6 pg/mL, no response to ACTH stim test, MRI normal | Not graded; life-threatening hyperkalemia | Hydrocortisone, sodium bicarbonate, insulin/glucose, calcium polystyrene sulfonate | Recovered, discharged in 18 days with ongoing hydrocortisone | Unique case of IAD with severe hyperkalemia; risk factors: CKD, T2D, ileal conduit, ARB use |
| Ohara et al., 2018 [22] | Advanced lung adenocarcinoma (LAC) | Nivolumab (3 mg/kg q2w) | 8 months after initiation (17th infusion) | Anorexia, fatigue, myalgia, severe hyponatremia | ACTH 3.1 pg/mL, cortisol 1.6μg/dL, no ACTH response to CRH, MRI: slight pituitary/stalk thickening | Not formally graded; consistent with central AI with MRI changes | Hydrocortisone 15 mg/day (oral), continued nivolumab | Improved symptoms and electrolytes, long-term replacement continued | HLA DRB1*04:05-DQA1*03:03-DQB1*04:01 genotype; suspected genetic susceptibility to polyglandular autoimmunity |
| Chen et al., 2024 [23] | Various (mainly lung cancer, melanoma, renal cancer) | Predominantly PD-1 inhibitors (nivolumab, pembrolizumab) | Median 8 cycles (range: 2 ~ 50); 8 cases after discontinuation (4~15 months) | Fatigue (80.95%), anorexia (52.38%), hypotension (32.14%), nausea/vomiting (30.95%) | ACTH deficiency (96.4%), MRI normal in 75.7%; pituitary enhancement in 14.9% | Not formally graded; 64.7% required hospitalization, 12.6% mortality (from FAERS) | Glucocorticoids (81/84), 62% physiologic/stress dose, 26% high-dose | Improved in most; only 2/24 had ACTH recovery | Largest analysis focusing on PD-1/L1-induced hypophysitis; delayed onset common, MRI often normal |
| Khatri et al., 2023 [24] | Unspecified lung cancer | Pembrolizumab | Within 1 week of last dose; ~7 weeks from initiation | Altered mental status, fatigue, hyponatremia | Cortisol 0.8μg /dL + 5.9 after cosyntropin; ACTH 11.8 pg/mL; TSH 74.95μIU/mL, FT4 0.51 ng/dL; MRI normal | Not formally graded; required hospitalization | Hydrocortisone →Levothyroxine | Improved, discharged; continued outpatient follow-up | Rare simultaneous presentation of secondary AI + primary hypothyroidism; emphasizes order of hormone replacement |
| Namikawa et al., 2021 [25] | Advanced gastric cancer (Stage IV) | Nivolumab (3 mg/kg q2w) | After 4 cycles (~2 months) | Fatigue, appetite loss | Cortisol 0.565μg /dL, ACTH 3.4 pg/mL; no response to CRH stimulation; MRI normal | Grade 3 irAE (described in text) | Hydrocortisone 20 mg/day orally | Improved; nivolumab continued; partial response (95% tumor shrinkage) | First English report of nivolumab-induced IAD in gastric cancer; pituitary stimulation test used for diagnosis |
| Antoniou et al., 2021 [26] | Skin melanoma | Nivolumab (21 cycles) | 6 months after discontinuation | Syncope, fatigue, mild anorexia, persistent hyponatremia (Na 123-125) | ACTH 1.0 pg/mL, cortisol 0.5 - 3.2μg/dL after ACTH test; MRI: thickened stalk & mild pituitary enlargement | Not graded; symptoms required hospitalization | Hydrocortisone 30 mg/day; emergency kit provided | Improved symptoms and electrolytes; discharged on steroid therapy | Rare case of delayed-onset hypophysitis after ICI withdrawal; thickened stalk observed despite normal prior MRI |
| Deligiorgi et al., 2020 [27] | Metastatic rectal adenocarcinoma (MSI-H, dMMR) | Nivolumab (240 mg q2w) | 16 weeks after initiation (1 month after 6th dose) | Profound fatigue, anorexia, disorientation, hypotension, palmar hyperpigmentation, hyponatremia (Na 125) | Cortisol 3μg /dL, ACTH 130-150 pg/mL, Synacthen test (8μg/dL at 60min), anti-21-hydroxylase Ab (+), CT: normal adrenal glands | Adrenal crisis requiring hospitalization; grade 3→4 equivalent | Hydrocortisone IV stress dose → oral tapering, no fludrocortisone needed | Complete recovery of adrenal function at 12 weeks; HC discontinued | First case of reversible ir-primary adrenal insufficiency (PAI) with positive anti-21-hydroxylase antibodies; aldosterone preserved |
| Escaño et al., 2024 [28] | Metastatic squamous cell carcinoma of the skin (lung metastases) | Pembrolizumab (q6w for 1 year) | Approximately 12 months after initiation | Fatigue, nausea, appetite loss, orthostatic hypotension, lightheadedness | Cortisol 1.1μg/dL, ACTH <3 pg/mL; MRI not yet performed | Not stated; hospitalization required | Solu-Medrol 20 mg IV x2 → oral hydrocortisone | Symptom resolution; discharged on steroids with outpatient pituitary imaging pending | Case emphasizes delayed recognition due to vague symptoms; thyroid labs suggest subclinical hypothyroidism |
| Atkins & Ur, 2020 [29] | Metastatic renal cell carcinoma | Ipilimumab | Not specified; occurred during treatment | Headache, visual disturbance, fatigue, nausea, hypotension | ACTH deficiency; MRI normal | Not formally graded; symptoms resolved, dysfunction persisted | Observation and hormone replacement | Imaging and symptoms resolved; AI persisted | Managed conservatively without high-dose steroids; pituitary dysfunction persisted |
| Atkins & Ur, 2020 [29] | Metastatic melanoma | Ipilimumab | Not specified; occurred during treatment | Headache, fatigue, libido loss, ED | Panhypopituitarism, stalk enlargement on CT | Not graded; hormone replacement required | High-dose steroids, discontinued ICI | Headaches and MRI findings improved; dysfunction persisted | Panhypopituitarism permanent despite therapy; thyroid, cortisol, testosterone replaced |
| Kanie et al., 2018 [30] | Stage IV NSCLC | Atezolizumab | After 56 weeks (19 doses) | General malaise, appetite loss, diarrhea, eosinophilia (14%) | ACTH 3.5 pg/mL, cortisol 0.2μg/dL, poor response to insulin tolerance test, MRI: anterior pituitary atrophy | Not stated; significant symptoms requiring admission | Hydrocortisone 15 mg/day | Rapid symptom improvement, continued ICI therapy, ACTH deficiency persists after 24 months | Anterior pituitary atrophy on MRI; isolated ACTH deficiency |
| Kanie et al., 2018 [30] | Stage IV NSCLC | Atezolizumab | After 52 weeks (18 doses) | Appetite loss, general malaise, eosinophilia (7.5%) | ACTH 13.7 pg/mL, cortisol 4.9μg/dL, poor ACTH/cortisol response, MRI: no abnormalities | Not stated; symptoms resolved with steroids | Hydrocortisone 15 mg/day | Improved; ACTH deficiency persists after 13 months | MRI normal; isolated ACTH deficiency without other pituitary axis involvement |
| Zilberman et al., 2023 [31] | BRCA-positive metastatic castration-resistant prostate cancer | Pembrolizumab | 8 months after discontinuation (after ~20 months of therapy) | Syncope, fatigue, nausea, vomiting, appetite loss, abdominal pain | Cortisol 0.34μg/dL, ACTH 3.9 pg/mL; no response to cosyntropin; MRI not performed | Not graded; clinical adrenal crisis requiring IV steroids | IV methylprednisolone → oral hydrocortisone taper; resumed levothyroxine | Marked improvement; returned to baseline; on HC 10 mg BID | Delayed-onset central adrenal insufficiency after ICI discontinuation; suggests need for long-term endocrine follow-up |
| Seki et al., 2017 [32] | Metastatic renal cell carcinoma | Nivolumab (3 mg/kg q2w) | After 6th dose (~12 weeks) | Fatigue, appetite loss, nausea, mild hyponatremia, eosinophilia | CRH & ACTH tests: isolated ACTH deficiency; MRI: normal pituitary | Not graded; symptoms resolved with HC | Hydrocortisone 10 mg BID | Improved fatigue and hyponatremia; long-term replacement continued | MRI normal despite pituitary dysfunction; example of PD-1-induced isolated ACTH deficiency without enlargement |
| Okano et al., 2016 [33] | Advanced malignant melanoma | Nivolumab (2 mg/kg q3w) | 1 week after 6th dose (~18 weeks) | Fatigue, appetite loss, thirst, hyponatremia, eosinophilia | ACTH 4.9 pg/mL, cortisol 1.7μg/dL, CRH/TSH test: isolated ACTH and delayed TSH response; MRI: mild pituitary/stalk enlargement | Not formally graded; compatible with Grade 2→3 | IV hydrocortisone 100 mg/day → oral taper (20 mg/day) | Symptom resolution; MRI normalized; continued nivolumab with monitoring | First detailed Japanese case with stalk enlargement and hormone axis testing; no anti-pituitary Ab |
| Rai et al., 2020 [34] | Metastatic renal cell carcinoma | Nivolumab (6 months) | 2 weeks after last dose | Fatigue, weakness, dizziness, refractory hypotension (BP 78/44), hyponatremia (Na 128) | Morning cortisol 1.3μg/dL, ACTH <5 pg/mL, post-cosyntropin cortisol 10.2μg/dL; MRI not performed | Not graded; required hospitalization for adrenal crisis | IV hydrocortisone → oral taper | Improved symptoms and BP; discharged on oral HC; nivolumab temporarily discontinued | Classic delayed-onset secondary adrenal insufficiency with hemodynamic compromise; underscores need for early suspicion |
| Shrotriya et al., 2018 [35] | Advanced NSCLC (Stage IV lung adenocarcinoma) | Nivolumab (4 cycles, completed 7 months before onset) | 7 months after discontinuation | Fatigue, malaise, weight loss (13.6 kg), hypotension, dry mucosa, low glucose | AM cortisol 0.4μg/dL, ACTH <5 pg/mL, no response to cosyntropin; MRI: normal pituitary | Grade 4 (hemodynamic instability requiring inpatient care) | HC 20 mg AM / 10 mg PM, fludrocortisone 0.1 mg for hypotension (discontinued later) | Rapid symptom resolution; able to return home; ACTH deficiency persisted | Classic delayed-onset isolated ACTH deficiency post-nivolumab; stresses generalist recognition of irAE |
| Ikeda et al., 2023 [36] | Unresectable hepatocellular carcinoma (HCC) | Atezolizumab + Bevacizumab | After 9 cycles | Fatigue, appetite loss, eosinophilia (15%), hyponatremia (Na 122), low glucose (75) | ACTH 2 pg/mL, cortisol 0.2μg/mL; poor response to ACTH, CRH, TRH; other hormones preserved; MRI: normal | Not graded; moderate symptoms with hospitalization | Hydrocortisone 15 mg/day | Rapid improvement of symptoms and labs; ICI resumed for 3 cycles before disease progression | First case in HCC with documented CRH, TRH, LHRH tests; early detection led to successful temporary ICI resumption |
| Ikeda et al., 2023 [36] | Unresectable hepatocellular carcinoma (HCC) | Atezolizumab + Bevacizumab | 3 months after ICI discontinuation (due to inflammatory arthritis) | Fatigue, appetite loss, eosinophilia (10.7%), hyponatremia (Na 124), low glucose (85) | ACTH 2.0 pg/mL, cortisol 2.9μg/mL; poor response to GHRP-2; MRI: normal | Not graded; symptoms required steroid initiation | Hydrocortisone 15 mg/day | Rapid symptom and biochemical improvement; unable to resume ICI due to poor performance status | Delayed-onset isolated ACTH deficiency after ICI cessation; eosinophilia may be early warning |
| Furuichi et al., 2023 [37] | Hepatocellular carcinoma (post-HCV cirrhosis) | Atezolizumab + Bevacizumab | After 13 cycles (~7 months) | Fatigue, appetite loss, muscle weakness, eosinophilia, hyponatremia | ACTH and cortisol decreased; CRH/TRH/GnRH stimulation: isolated ACTH deficiency; MRI: normal | Not stated; significant symptoms requiring treatment | Hydrocortisone (unspecified dose) | Rapid improvement, therapy continued (22 cycles) | Retrospective eosinophilia and Na drop preceded symptoms; early detection critical |
| Furuichi et al., 2023 [37] | HCC (NASH-related) | Atezolizumab + Bevacizumab | After 8 cycles | Fatigue, appetite loss, seizures, hyponatremia, eosinophilia | ACTH and cortisol decreased; MRI: normal | Likely grade 3→4; seizures, hypotension, hospitalization | Hydrocortisone | Improved; therapy continued (partial response) | ACTH/cortisol decline evident in retrospective labs before symptom onset |
| Furuichi et al., 2023 [37] | HCC (alcoholic liver disease) | Atezolizumab + Bevacizumab | After 10 cycles (at hospitalization for lenvatinib start) | Fatigue, appetite loss, hyponatremia, hypotension | ACTH and cortisol decreased; TSH ↑, FT4 ↓ (primary hypothyroidism); pituitary CT: normal | Likely grade 3 | Hydrocortisone + levothyroxine | Improved; switched to lenvatinib under steroid coverage | Only case with concurrent hypothyroidism; hormone replacement effective |
| Hata et al., 2021 [38] | Malignant mesothelioma | Nivolumab (240 mg q2w) | After 12 courses (with symptoms starting post-8th course, ~4 months) | Mild abdominal pain (initial), then fatigue, hyponatremia, hypotension | ACTH 0.75 pmol/L, cortisol 34.5 nmol/L (~1.25μg/dL), CRH test: no response, MRI: normal | Grade 3→4 equivalent (hemodynamic instability, hospitalization) | Oral hydrocortisone 15 mg/day | Rapid improvement; discharged on HC, Na normalized; ICI discontinued | First case of nivolumab-induced IAD presenting with abdominal pain; emphasizes abdominal pain + hyponatremia as potential AI clues |
| Takaya et al., 2017 [39] | Metastatic lung adenocarcinoma (Stage IV) | Nivolumab (3 mg/kg q2w) | 2 days after 12th dose (~5.5 months) | Fatigue, appetite loss, low BP (88/59), mild fever, dry mucosa | ACTH <1.0 pg/mL, cortisol 0.4μg/dL; CRH test: no ACTH/cortisol response; MRI: normal | Grade 3→4 (hemodynamic instability, hospitalization) | IV hydrocortisone 150 mg/day → oral 15 mg/day | Rapid improvement, normalization of Na/TSH/PRA; ACTH deficiency persisted, ICI discontinued | Well-characterized case with hormone stimulation tests and follow-up; ACTH and cortisol remained low |
| Gaballa et al., 2020 [40] | Metastatic melanoma (lung and cervical nodes, unknown primary) | Ipilimumab (4 cycles), followed by Nivolumab (16 cycles) | After 4 cycles of ipilimumab | Fatigue, weakness, nausea, abdominal pain, dizziness, weight loss, hypotension, mucosal pigmentation | Cortisol 5- 6.8/10.2μg/dL post-cosyntropin, ACTH 120 pg/mL, undetectable aldosterone, high renin (31 ng/mL/h); MRI: no hypophysitis | Grade 3 (hospitalization, functional limitation) | Hydrocortisone 100 mg IV bolus → 50 mg IV q6h, then taper | Symptomatic improvement within 24h; discharged with oral taper; full tumor remission after 16 cycles nivolumab | Clear-cut primary adrenal insufficiency (PAI) induced by ipilimumab; ACTH and renin elevated, aldosterone undetectable; no pituitary involvement |
